# Peritoneal lymphoma with ascites mimicking portal hypertensive ascites: A case report

**DOI:** 10.1097/MD.0000000000014583

**Published:** 2019-02-22

**Authors:** En-Shao Liu, Jyh-Seng Wang, Wen-Chi Yang

**Affiliations:** a Department of Internal Medicine; b Department of Pathology and Lab Medicine, Kaohsiung Veterans General Hospital; c Division of Hematology and Medical Oncology, Department of Internal Medicine, E-Da Hospital; d I-Shou University, Kaohsiung, Taiwan.

**Keywords:** ascites, lymphoma, peritoneum

## Abstract

**Rationale:**

Lymphoma with an initial manifestation of ascites and peritoneal invasion is rare.

**Patient concerns:**

A 65-year-old woman presented to the emergency department with a 3-week history of abdominal distention, anorexia, and night sweating, and a 2-week history of melena. She was a silent hepatitis B virus carrier. Abdominal ultrasound showed massive ascites without cirrhosis. Abdominal computed tomography revealed ascites, infiltrative peritoneal lesions with omental cake appearance, and lymphadenopathies.

**Diagnosis:**

We performed paracentesis and the ascites cytology was obtained. The patient also underwent esophagogastroduodenoscopy, which showed ulcerative tumors in the stomach. Both ascites cytology and pathology of the gastric tumors confirmed the diagnosis of B-cell lymphoma.

**Interventions:**

This patient received 7 cycles of chemotherapy.

**Outcomes:**

Follow-up imaging studies revealed partial remission of lymphoma, but an enlargement of residual tumors in omentum and mesentery, which resulted in intractable ascites and rapid deterioration of performance status. Despite a change of regimen of chemotherapy, this patient expired 10 months after diagnosis.

**Lessons:**

Lymphoma should be one of the differential diagnoses in patients with intractable ascites not attributable to other comorbidities.

## 1 Introduction

Primary extranodal lymphoma occurs in approximately 25% to 40% of patients with lymphoma and is more common in patients with non-Hodgkin lymphoma (NHL). Diffuse large B-cell lymphoma (DLBCL) is the dominant histological subtype of primary extranodal lymphoma.^[1]^ It is often intermediate to high grade and indicates a poor prognosis.^[2,3]^

The gastrointestinal tract is the most frequent extranodal site of lymphoma, accounting for about 40% of cases. In the gastrointestinal tract, the stomach is the most common involved site, followed by the small intestine and ileocecal region.^[1,4]^ Malignant ascites indicates the presence of cancer cells in the peritoneal cavity and is an ominous prognostic sign. Tumors causing malignant ascites are more commonly from peritoneal surface malignancies such as ovaries, colorectum, and pancreas. Peritoneal lymphomatosis (PL), defined as the peritoneal spreading of lymphoma, is rare and receives much less attention. Lymphoma does not usually invade the omentum, because it is fibro-fatty in nature and lacks lymphoid tissue.^[5]^ It is important to differentiate PL from carcinomatosis, which commonly invades from the stomach, colon, or ovaries, as they require different chemotherapeutic intervention and have different potential curability.^[6]^ Here, we share a case of gastric DLBCL with an initial presentation of massive ascites. Informed consent was obtained from the patient and her family for the publication of this case report.

## 2 Case presentation

**Figure 1:**
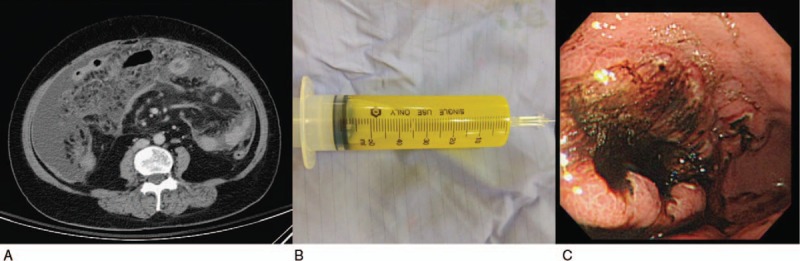
Abdominal computed tomography and esophagogastroduodenoscopy images showing ascites. (A) Diffuse lymphomatous infiltration of the omentum, causing peritoneum thickening. (B) Straw-colored ascites, without blood or chyle. (C) Infiltration of ulcerative gastric mass with coffee-ground material.

**Figure 2:**
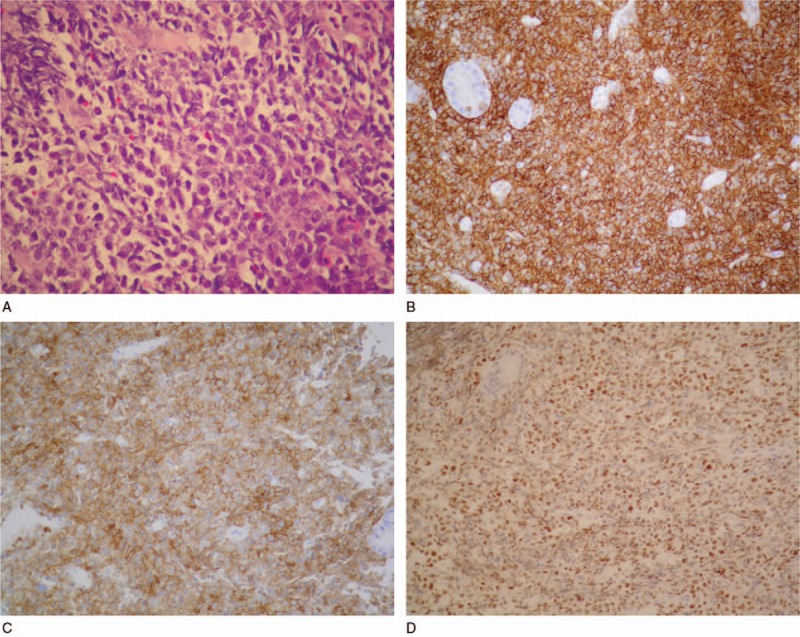
Pathology and immunochemistry of gastric tumors. (A) Hematoxylin–eosin staining. (B) Immunochemical staining showing CD20 positivity. (C) Immunochemical staining showing CD10 positivity. (D) Immunochemical staining showing Bcl-6 positivity.

**Figure 3:**
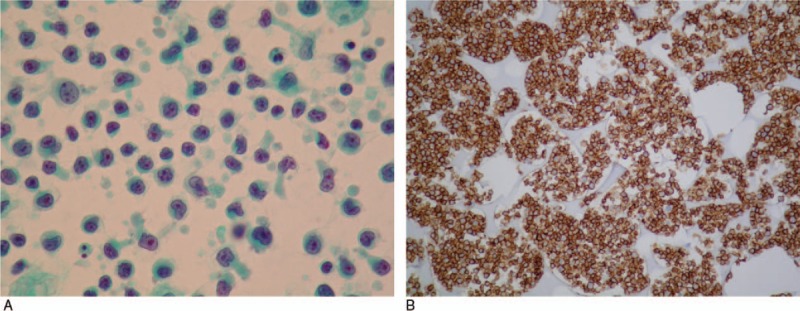
Ascites cytology. (A) Microscopic findings of ascites cytology. (B) Immunochemical staining of ascites cytology plus cell block showing CD20 positivity.

**Figure 4:**
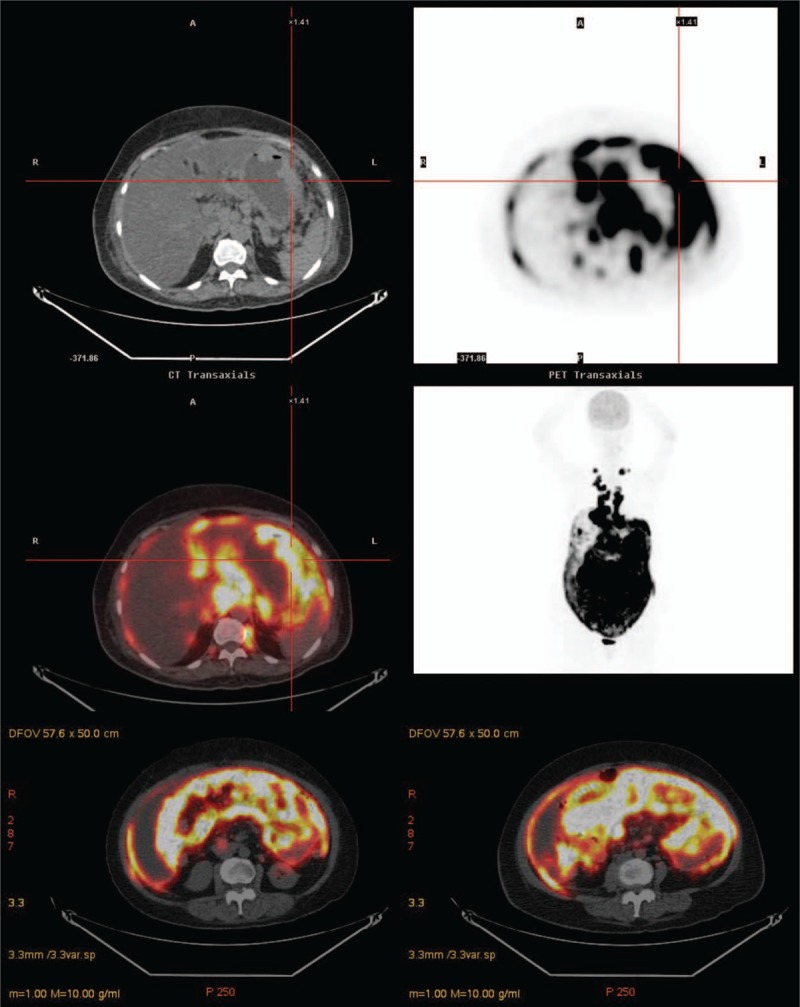
PET scan showing intense FDG uptake in the omentum and stomach corresponding to peritoneal lymphomatosis and stomach invasion. PET = positron emission tomography, FDG = fluorodeoxyglucose.

A 65-year-old woman presented to our hospital after experiencing rapid abdominal distension in 3 weeks. Additional symptoms included fatigue, anorexia, and night sweating. There was no other discomfort, except melena in the past 2 weeks. She was a silent hepatitis B virus carrier and neither smoked nor consumed alcohol. She had retired from a carton factory several years previously. Physical examination revealed abdominal distension with shifting dullness. Neither lymphadenopathy nor hepatosplenomegaly was detected. Laboratory studies showed the following data: white blood cells (WBCs), 10400/μL; neutrophils, 60.0%; lymphocytes, 24%; monocytes, 14%; hemoglobin, 11.4 g/dL; mean corpuscular volume, 91.4 fL; platelets, 417,000/μL; albumin, 3.4 g/dL; lactate dehydrogenase (LDH), 901 U/L; and β2-microglobulin, 3861 ng/mL. No other electrolytes or renal or liver function abnormalities were noted. Tumor marker examination revealed increased CA-125 (425.8 U/mL); CEA, CA-199, CA-153, and tissue polypeptide antigen were within the normal limits. She had previously undergone total hysterectomy and bilateral oophorectomy, which were confirmed by transabdominal sonography. Computed tomography (CT) revealed massive ascites and peritoneal lesions with omental cake, along with metastatic paraaortic and paradiaphragmatic lymph nodes (Fig. 1A). Paracentesis for ascites analysis was performed, and it was yellow and clear in appearance (Fig. 1B). Other lab data of ascites included the following: WBCs, 54000/mm^3^; red blood cells, 720/mm^3^; neutrophils, 1%; lymphocytes, 76%; monocytes, 23%; LDH, 1415 U/L; protein, 3.1 g/dL; albumin, 1.7 g/dL; serum-ascites albumin gradient (SAAG), 1.4; sugar, 10 mg/dL; and amylase, 25 U/L. No microorganisms were detected, and acid-fast stain of ascites was negative. Ascites cytology plus cell block was also obtained. She underwent esophagogastroduodenoscopy because of melena, and it showed a large confluent infiltrative lesion about 10 cm in length with several ulcerations and a giant fold at the greater curvature side of the lower body of the stomach. Another lesion 3 cm in size with ulceration at the high body was also discovered, along with much coffee-ground material (Fig. 1C). A submucosal lesion, such as lymphoma or gastric adenocarcinoma, Borrmann classification type IV, was suspected. The pathology of the gastric tumors revealed diffuse infiltration of large atypical lymphocytes with centroblast features, and immunohistochemistry was positive for CD20, Bcl-2, CD10, MUM1, Bcl6, and c-MYC, and negative for CD3, which confirmed the diagnosis of DLBCL, germinal center B-cell type (Fig. 2A–D). Immunohistochemistry of the ascites cell block showed malignant cells positive for CD20 and negative for CD3, D2–40, Ber-EP4, and MOC-31, which was compatible with B-cell lymphoma (Fig. 3Aand B). Bone marrow biopsy was also performed, and the pathological examination revealed bone marrow involvement. We performed positron emission tomography (PET) for staging, which revealed diffusely nodal involvement in the bilateral infraclavicular/internal mammary chains, paraaortic/paradiaphragmatic regions, stomach, omentum, and mesentery (Fig. 4). According to these results, gastric DLBCL, stage IV was diagnosed. The patient then received rituximab, cyclophosphamide, doxorubicin, vincristine, and prednisone for lymphoma treatment. Notably, under the concern of HBV infection, we prescribed entecavir before chemotherapy for prophylaxis of HBV flare-up. After 7 courses of treatment, a follow-up PET scan demonstrated partial remission of lymphoma, but an enlargement of residual tumors in omentum and mesentery as compared with the initial studies 8 months ago. During preparation for autologous peripheral blood stem cell transplantation, this patient's performance status deteriorated rapidly with intractable ascites. The treatment regimen was shifted to rituximab and bendamustine, followed by lenalidomide. Sepsis and acute kidney injury occurred after 1 course of therapy, and the patient expired 10 months after diagnosis.

## 3 Discussion

Lymphomas presenting with serous effusion are common, but the prevalence varies in different cavities. The frequency of pleural effusion is 20% to 30% in NHL and Hodgkin disease; however, ascites is a rare presentation of lymphomas.^[7]^ According to Vakar-Lopez and Yang, only 20 cases of lymphoma with ascites as the initial presentation have been reported.^[8]^ In a series of 101 cytologically proven malignant ascites cases, Runyon and Hoefs observed lymphoma (NHL) in only 8 cases (8%).^[9]^ In another analysis of 100 patients with ascites, Mahmood et al reported lymphoma as the underlying cause of only 2% of cases.^[10]^

Malignant lymphoma can present with peritoneal invasion in 2 ways. First, a primary peritoneal lesion, also known as primary effusion lymphoma, is a rare type of NHL presenting with serous effusions without detectable tumor formation. It occurs in immunocompromised patients and is related to KSHV/HHV-8 infection and coinfection with Epstein-Barr virus or HIV.^[7,11,12]^ Second, the peritoneal invasion continuously spreads from an extranodal lymphoma of intra-abdominal origin. In a study of 400 patients with newly diagnosed or recurrent NHL, Glazer et al reported that the peritoneum and omentum were involved in 7 patients (1.75%), and ascites was observed in 4 patients (1%).^[13]^ However, development of serous effusion in the course of lymphomas, either primary or otherwise, is considered an adverse factor affecting overall survival.

Paracentesis is a safe, cost-effective, useful tool that should be considered as the first-line evaluation to survey ascites. The SAAG, which was first proposed by Hoefs et al in 1981,^[14]^ is calculated by subtracting the ascites albumin concentration from the serum albumin concentration. This is a more sensitive and specific measure for the differentiation of ascites due to portal hypertension from ascites due to other pathophysiological mechanisms (eg, peritoneal inflammation).^[15]^ Peritoneal malignancy without overt liver masses typically has a fluid WBC count of 500/mm^3^ or greater, a SAAG of less than 1.1 g/dL, and total protein of 2.5 g/dL or greater.^[16,17]^ In this patient, omentum cakes and thickening with positive malignant cells in the ascites confirmed malignant ascites, and neither liver problems nor cardiac diseases were found. Therefore, it is unusual that she also had malignant ascites presenting with a SAAG greater than 1.1 g/dL, which is more common in portal hypertension-related ascites. This may be because peritoneal lymphoma changes the permeability of peritoneal vessels and leads to fluid extruding from the vessels to the peritoneal cavity. Distinguishing between peritoneal carcinomatosis and PL is challenging but important, because their respective treatments and prognoses are quite different. CT findings of omental caking with homogeneous bulky masses, rather than a nodular pattern, diffuse lymph node involvement, splenomegaly, and the presence of tumors in the gastrointestinal tract, especially the stomach and terminal ileum, have been proposed to support a diagnosis of PL.^[17,18]^ However, most patterns of tumor involvement are similar, and a definitive diagnosis of PL is impossible from imaging alone. The appearance of ascites can also provide some clues in the differential diagnosis. In a small analysis, approximately 20% of malignant ascites samples were bloody, and hepatocellular carcinoma was the underlying cause of the majority of bloody ascites. In contrast, lymphoma-associated bloody ascites was rarely reported.^[19]^ Chylous ascites is a rare form of ascites resulting from disruption of the lymphatic system. Milky appearing ascites with a triglyceride concentration above 200 mg/dL is diagnostic; abdominal malignancy, cirrhosis, and tuberculosis are the major causes in adults. Intra-abdominal solid organ tumors, such as malignancies in the stomach, esophagus, pancreas, endometrium, and prostate, account for 40% of all malignant ascites.^[20]^ However, the appearance of the ascites in our case was yellow and transparent, similar to the appearance of ascites due to cirrhosis or portal hypertension.

The relation between lymphoma and elevated serum LDH level is well established. In addition to being used for diagnosis, the LDH level may be an independent prognostic factor of lymphoma.^[21]^ Ascites LDH has high sensitivity and low specificity for malignant ascites. Kim et al reported that the ascites LDH level is elevated in a few PL patients, supporting the idea that the ascites LDH level can be an important clue for differential diagnosis.^[6]^ In our case, the presentations of elevated protein and LDH levels in the ascites, low glucose concentration, and sterile ascites were compatible with the clinical features of PL.

Serum CA-125 is a mucin-like glycoprotein antigen expressed in normal tissue originally derived from celomic epithelium, such as the peritoneum, pleura, fallopian tubes, and endometrium. It is elevated in ovarian cancer, but is not necessarily indicative of malignancy.^[22]^ Since elevated serum CA-125 can be found in patients with ascites or pleural effusion of any etiology, it is presumed to result from the shear force on mesothelial cells. Extensive peritoneal or omental lymphomatosis might lead to elevated serum CA-125.^[23]^ Our patient had increased CA-125, but she had previously undergone bilateral oophorectomy, and there were no gynecological lesions noted during sonography and physical examination studies. Evaluation combined with findings of ascites cells and CT scan suggested that the elevated serum CA-125 was related to PL.

Ascites cytology is important to diagnose malignant ascites. However, the sensitivity rate is affected by the number and quality of specimens processed. Review of the current literature suggests the overall sensitivity of cytology is around 60% to 70%.^[24,25,26]^ The cause of malignant ascites may also relate to the sensitivity. Lynch et al reported a series of 7 patients with NHL and PL where the cytology provided diagnosis in only 1 patient.^[27]^ Moreover, lymphoma can elicit florid mesothelial hyperplasia, confusing the cytology results and extending the diagnostic process. Therefore, if possible, histology is still the gold standard for diagnosis.

In conclusion, we provide a gastric lymphoma case of PL showing portal hypertension mimic ascites with high SAAG at initial presentation. PL is a rare presentation that mimics peritoneal carcinomatosis, but is different in terms of treatment and prognosis. Multidisciplinary evaluation of history, imaging findings, ascites analysis, and especially cytology should be performed to ensure proper diagnosis.

## Author contributions

**Conceptualization:** Wen-Chi Yang.

**Data curation:** Jyh-Seng Wang.

**Supervision:** Wen-Chi Yang.

**Visualization:** Jyh-Seng Wang.

**Writing – original draft:** En-Shao Liu.

**Writing – review and editing:** Wen-Chi Yang.
